# National inventory of authorized diagnostic imaging equipment in Ghana: data as of September 2020

**DOI:** 10.11604/pamj.2022.41.301.30635

**Published:** 2022-04-14

**Authors:** Bright Kwadwo Bour, Edem Kwabla Sosu, Francis Hasford, Prince Kwabena Gyekye, Daniel Gyingiri Achel, Augustine Faanu, Joseph Kwabena Amoako, Richard Denys Pitcher

**Affiliations:** 1 School of Nuclear and Allied Sciences, University of Ghana, Accra, Ghana,; 2 Radiological and Medical Sciences Research Institute, Ghana Atomic Energy Commission, Accra, Ghana,; 3 Radiological and Non-Ionizing Radiation Directorate, Nuclear Regulatory Authority, Accra, Ghana,; 4 Radiation Protection Institute, Ghana Atomic Energy Commission, Accra, Ghana,; 5 Division of Radiodiagnosis, Faculty of Medicine and Health Sciences, Stellenbosch University, Cape Town, South Africa

**Keywords:** Diagnostic radiology, medical imaging, equipment, population ratio, Ghana

## Abstract

**Introduction:**

to address the challenge of inadequate and non-equitable distribution of diagnostic imaging equipment, countries are encouraged to evaluate the distribution of installed systems and undertake adequate monitoring to ensure equitability. Ghana´s medical imaging resources have been analyzed in this study and evaluated against the status in other countries.

**Methods:**

data on registered medical imaging equipment were retrieved from the database of the Nuclear Regulatory Authority and analyzed. The equipment/population ratio was mapped out graphically for the 16 regions of Ghana. Comparison of the equipment/population ratio was made with the situation in other countries.

**Results:**

six hundred and seventy-four diagnostic imaging equipment units from 266 medical imaging facilities (2.5 units/facility), comprising computed tomography (CT), general X-ray, dental X-ray, single-photon emission computed tomography (SPECT) gamma camera, fluoroscopy, mammography and magnetic resonance imaging (MRI) were surveyed nationally. None of the imaging systems measured above the Organization for Economic Co-operation and Development (OECD) average imaging units per million populations (u/mp). The overall equipment/population ratio estimated nationally was 21.4 u/mp. Majority of the imaging systems were general X-ray, installed in the Greater Accra and Ashanti regions. The regional estimates of equipment/population ratios were Greater Accra (49.6 u/mp), Ashanti (22.4 u/mp), Western (21.4 u/mp), Eastern (20.6 u/mp), Bono East (20.0 u/mp), Bono (19.2 u/mp), Volta (17.9 u/mp), Upper West (16.7 u/mp), Oti (12.5 u/mp), Central (11.9 u/mp), Northern (8.9 u/mp), Ahafo (8.9 u/mp), Upper East (6.9 u/mp), Western North (6.7 u/mp), Savannah (5.5 u/mp) and North-East (1.7 u/mp).

**Conclusion:**

medical imaging equipment shortfall exist across all imaging modalities in Ghana. A wide inter-regional disparity in the distribution of medical imaging equipment exists contrary to WHO´s recommendation for equitable distribution. A concerted national plan will be needed to address the disparity.

## Introduction

Diagnostic imaging has become an important component of healthcare delivery and has impacted on life expectancy worldwide. In 2010, Welling *et al*. demonstrated the use of radiological services for the assessment, diagnosis and monitoring of treatment of at least 30% of patients with varied medical conditions [1]. Given the increased popularity and usage of diagnostic radiology across the world [2-4], there is growing need to attach importance to the establishment of diagnostic imaging infrastructure to ensure national and world-wide equitable access [5]. The World Health Organization (WHO) recommends one basic X-ray and one ultrasound unit for 50,000 people, which is expected to meet 90% of global imaging needs [6]. The WHO and Organization for Economic Co-operation and Development (OECD) have records on national estimates of high-end radiology equipment resources based on questionnaire surveys of member states [7,8].

Mollura and Lungren [9] estimated that two-thirds of the world´s population have no access to basic medical imaging services. The 60^th^ World Health Assembly of the United Nations held in May 2007, adopted a resolution for member states to collect, verify, update and exchange information on health technologies, with particular emphasis on medical devices [10]. Ghana, being a United Nations (UN) member state, has taken some initiatives in this regard but is yet to take inventory of registered diagnostic imaging equipment.

The scope of diagnostic imaging procedures in Ghana covers general radiography (X-ray), dental X-ray, mammography, computed tomography (CT), fluoroscopy, interventional radiology, magnetic resonance imaging (MRI), ultrasound and nuclear medicine. Studies which present picture of the distribution of the facilities across Ghana is lacking, unlike in other African countries like South Africa [11], Zambia [12], Tanzania [13], Zimbabwe [14] and Uganda [15] where this study has been conducted. This study aimed to analyze registered diagnostic imaging equipment units in Ghana and evaluate their distribution as well as equipment/population ratios in all geographical regions of the country.

## Methods

**Study setting and period:** Ghana is a lower-middle income country (LMIC) [16] and has population of 31,478,523 and land area of 238,533 km^2^ as of June 2021. The country is characterized by a population pyramid with a wide base, a population growth rate of 2.2% per annum and a life expectancy of 66.6 years [17]. Diagnostic imaging infrastructure in Ghana is related to the country´s resources available to deal with the prevention, screening and early detection, diagnosis, treatment and palliative care for cancer control. The public sector is responsible for 61% of health service provision, followed by private sector (31%) and faith-based organizations (7%). Forty-five percent of the population is covered under one insurance or the other, with doctor-to-population ratio of 1: 8100 as of June 2021. Data for this inventory study were collated from October 2019 to September 2020 from the database of the Nuclear Regulatory Authority (NRA) of Ghana. The NRA maintains updated database of all licensed diagnostic imaging equipment in the country and their regional and district locations.

**Data source:** data on ionizing radiation equipment (general X-ray, dental X-ray, fluoroscopy, mammography, CT and Single Photon Emission Computed Tomography (SPECT) gamma camera) were retrieved from the Regulatory Authority Information System (RAIS) of the Nuclear Regulatory Authority (NRA), while data on MRI systems were obtained from the published research of Ofori *et al*. (2020) [18]. Ultrasound equipment was excluded, due to the absence of a national database and no published data on ultrasound units in the country.

**Statistical analysis:** the data were captured on Microsoft Excel spreadsheet and analyzed based on imaging modality types, regional distribution of equipment, equipment/population ratio (units per million people (u/mp)) and ownership. The 2020 projected human population data used for the survey analysis was obtained from the website of the Ghana statistical service [12], the body mandated to produce and disseminate official statistics in the country. The equipment density was mapped out for each of the 16 regions of Ghana and presented graphically to give a clear picture of the distribution of available diagnostic imaging facilities across the country. Comparative analysis of equipment/population ratios by imaging modality were also performed.

## Results

**Figure 1:**
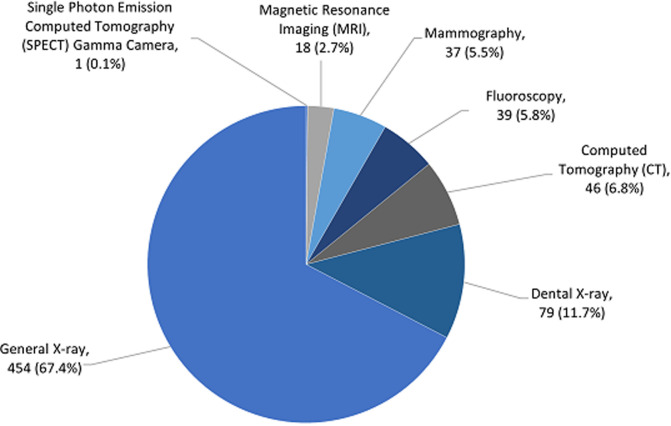
number of imaging equipment units in Ghana

**Figure 2:**
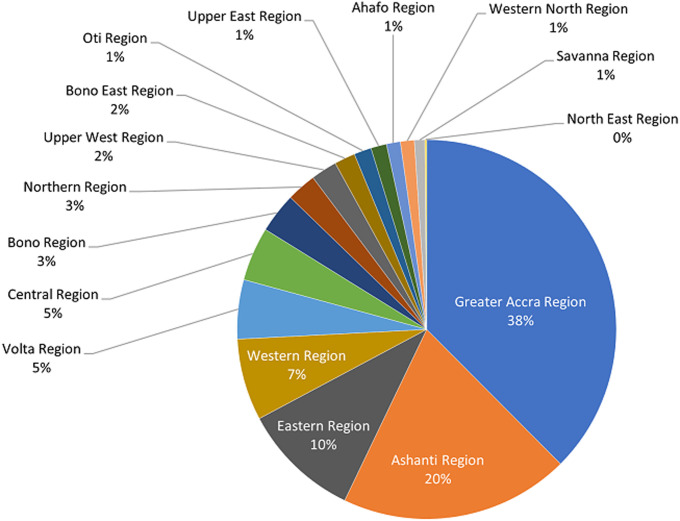
distribution of diagnostic imaging equipment in the 16 regions of Ghana

**Distribution of diagnostic imaging equipment in Ghana:** there are 674 diagnostic imaging equipment units from 266 medical facilities in Ghana, including the modalities general X-ray, dental X-ray, fluoroscopy, mammography, CT, MRI and SPECT machines. Figure 1 presents number of the imaging equipment units in the country as of September 2020, while Figure 2 shows their regional distribution. The results indicate 58% (n=674) of the imaging systems are concentrated in the Greater Accra and Ashanti regions of the country, with majority (67% (n=674)) of them being general X-ray systems. Number of the diagnostic healthcare centres in private ownership (52% (n=266)) are more than in state/public ownership (48% (n=266)). Greater Accra, among 15 other geographical regions of the country, is the only region with full spectrum of diagnostic imaging modalities considered under this survey. Three regions with more numbers of imaging units per centre than the national average of 2.5 (Table 1) are the Greater Accra, Eastern and Bono.

**Table 1:** equipment/diagnostic centre ratios

Region	Number of imaging units	Number of diagnostic medical centres			Unit(s) per centre ratio
		Public	Private	Total	
Greater Accra	253	26	59	85	3.0
Ashanti	132	20	35	55	2.4
Western	47	13	7	20	2.4
Eastern	68	14	9	23	3.0
Bono East	12	5	0	5	2.4
Bono	23	4	3	7	3.3
Volta	34	9	8	17	2.0
Upper West	15	4	2	6	2.5
Oti	10	5	1	6	1.7
Central	31	8	7	15	2.1
Northern	17	6	3	9	1.9
Ahafo	8	2	2	4	2.0
Upper East	9	3	1	4	2.3
Western North	8	5	1	6	1.3
Savannah	6	3	0	3	2.0
North-East	1	1	0	1	1.0
Total (national)	674	128	138	266	2.5

**Figure 3:**
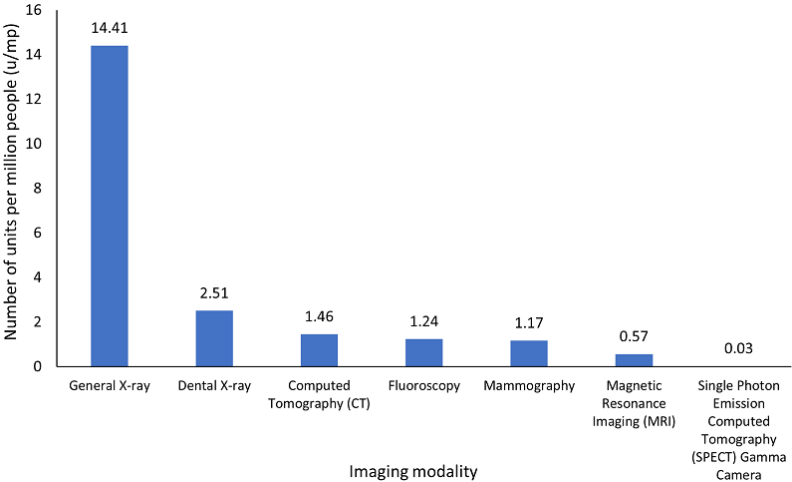
units per million people by imaging modality in Ghana

**Figure 4:**
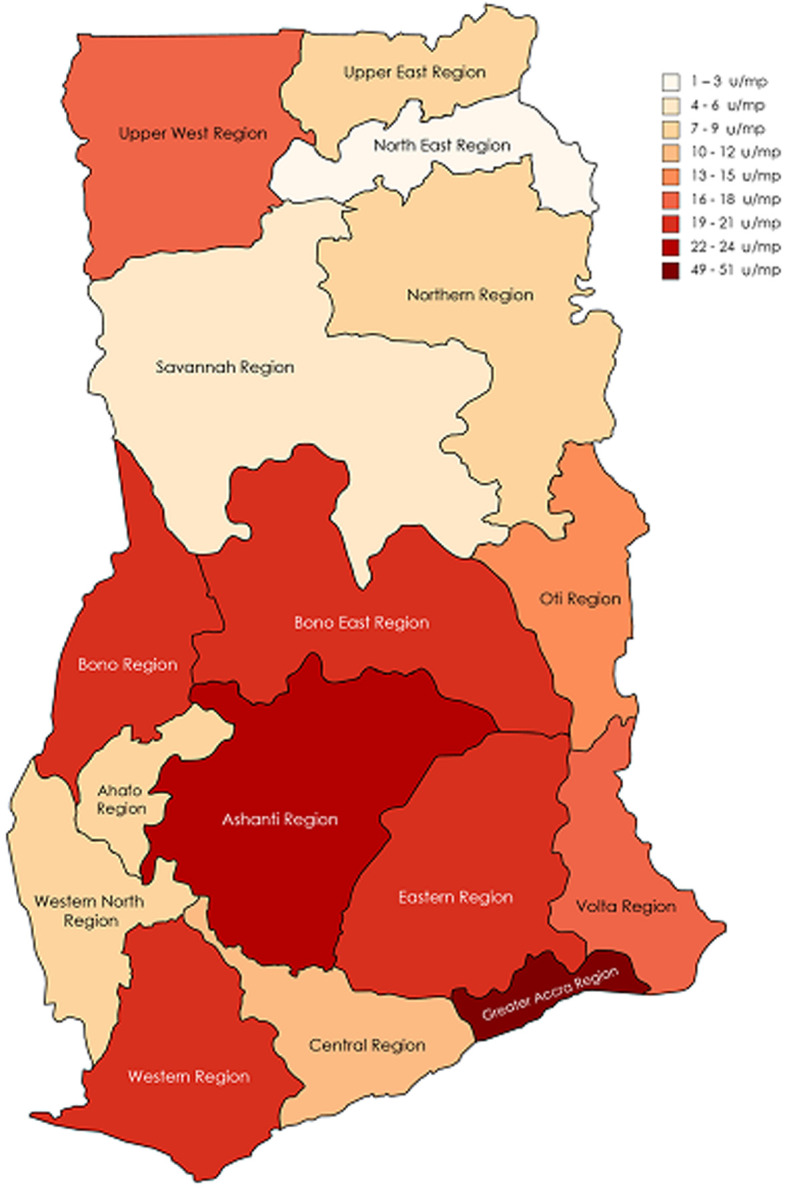
geographical regional map of Ghana showing imaging equipment/population ratios

**Equipment to population ratios:** the equipment/population ratios (units per million people) by imaging modality and geographical region are presented in Figure 3 and Figure 4 respectively. General X-ray, dental, CT, fluoroscopy, mammography, MRI and SPECT systems have population ratios of 14.41, 2.51, 1.46, 1.24, 1.17, 0.57 and 0.03 respectively. The Greater Accra with imaging units/population ratio of 49.6 u/mp and Ashanti with 22.4 u/mp are the two most dense equipment distribution regions in the country. Detailed data are presented in Table 2. The study reveals that, general X-ray equipment is the most common medical imaging device in use locally while SPECT gamma camera system is the least popular.

**Table 2:** regional breakdown of diagnostic imaging equipment per million population

Region	Population (X106)	Area (X103 km^2^)	Population density (X103 people/km^2^)	General X-ray		Dental X-ray		CT^1^		Fluoroscopy		Mammography		MRI^2^		SPECT^3^ gamma camera		Total	
				n	u/mp	n	u/mp	n	u/mp	n	u/mp	n	u/mp	n	u/mp	n	u/mp	n	u/mp
Greater Accra	5.1	3.2	1594	139	27.3	45	8.8	25	4.9	10	2.0	21	4.1	12	2.4	1	0.2	253	49.6
Ashanti	5.9	24.4	242	79	13.4	17	2.9	12	2.0	12	2.0	9	1.5	3	0.5	0	-	132	22.4
Western	2.2	13.8	159	40	18.2	2	0.9	3	1.4	1	0.5	1	0.5	0	-	0	-	47	21.4
Eastern	3.3	19.3	171	52	15.8	8	2.4	1	0.3	6	1.8	1	0.3	0	-	0	-	68	20.6
Bono East	0.6	23.3	26	10	16.7	0	-	1	1.7	1	1.7	0	-	0	-	0	-	12	20.0
Bono	1.2	11.1	108	16	13.3	3	2.5	1	0.8	2	1.7	1	0.8	0	-	0	-	23	19.2
Volta	1.9	9.5	200	30	15.8	0	-	0	-	2	1.1	1	0.5	1	0.5	0	-	34	17.9
Upper West	0.9	18.5	49	13	14.4	0	-	0	-	1	1.1	1	1.1	0	-	0	-	15	16.7
Oti	0.8	11.1	72	9	11.3	1	1.3	0	-	0	-	0	-	0	-	0	-	10	12.5
Central	2.6	9.8	265	23	8.8	2	0.8	1	0.4	3	1.2	1	0.4	1	0.4	0	-	31	11.9
Northern	1.9	25.4	75	12	6.3	0	-	2	1.1	1	0.5	1	0.5	1	0.5	0	-	17	8.9
Ahafo	0.9	5.2	173	8	8.9	0	-	0	-	0	-	0	-	0	-	0	-	8	8.9
Upper East	1.3	8.8	148	8	6.2	1	0.8	0	-	0	-	0	-	0	-	0	-	9	6.9
Western North	1.2	10.1	119	8	6.7	0	-	0	-	0	-	0	-	0	-	0	-	8	6.7
Savanna	1.1	35.9	31	6	5.5	0	-	0	-	0	-	0	-	0	-	0	-	6	5.5
North-East	0.6	9.1	66	1	1.7	0	-	0	-	0	-	0	-	0	-	0	-	1	1.7
Total	31.5	238.5	132	454	14.41	79	2.51	46	1.46	39	1.24	37	1.17	18	0.57	1	0.03	674	21.40

N: number of diagnostic imaging equipment; u/mp: units per million population; ^1^computed tomography; ^2^magnetic resonance imaging; ^3^single photon emission computed tomography

Comparative equipment/population ratios from this study and published international data from Africa [11-15] and OECD [19,20] countries are presented in Table 3. South Africa (5.03 u/mp) and Zimbabwe (1.50 u/mp) are the countries with better distribution of CT systems per million population relative to Ghana. South Africa consistently produced better equipment/population distribution of 4.96, 2.9, 34.8 and 6.60 for mammography, MRI, X-ray, fluoroscopy.

**Table 3:** equipment/population ratio comparisons

		Units per million people					
		CT	Mammography	MRI	General X-ray	Fluoroscopy	SPECT gamma camera
Ghana (this study)		1.46	1.17	0.57	14.41	1.24	0.03
Other African countries	Egypt			2.00			
	Kenya	0.80	0.50				
	South Africa	5.03	4.96	2.90	34.80	6.60	
	Tanzania	0.42	0.31	0.09	9.02	1.00	
	Uganda	0.60	0.50		9.60	0.80	
	Zambia	0.79	1.22	0.24	14.30	0.55	0.06
	Zimbabwe	1.50	0.80	0.50	26.00	0.80	
OECD data, 2015 or nearest year	Belgium		36.57				
	Brazil	15.34		6.76			
	Canada	15.01	18.05	9.48			
	Germany	35.09		33.63			
	Greece		65.97				
	Iceland	39.30	16.81	21.16			
	Israel	9.79		4.06			
	Italy	33.31	33.83	28.24			
	Japan	107.17	34.32	51.69			
	Korea	37.03	61.57	26.27			
	Lithuania	21.00	15.75	11.02			
	Mexico	5.92	9.73	2.39			
	New Zealand	17.84	19.70	13.27			
	United Kingdom	9.46		7.23			
	United States of America	40.98	65.26	39.00			
	OECD average	25.72	24.34	15.94			

CT: computed tomography; MRI: magnetic resonance imaging; SPECT: single-photon emission computed tomography; OECD: organization for economic co-operation and development

## Discussion

As a country, Ghana needs quality data on available medical imaging systems to inform the radiological healthcare needs of its inhabitants. This study provides a clear picture of the regional distribution of available diagnostic imaging systems in the country and their comparison, relative to recommendations of the WHO. The study provides analysis of the diagnostic imaging capacity in Ghana and as such adds significant new insights into the provision of radiological healthcare services in the country. Five of the regions (Greater Accra, Ashanti, Western, Eastern and Bono East) recorded equipment/population ratios exceeding 20 units per million people. However, on the national scale, equipment/population ratios are much lower than OECD averages for respective imaging modalities. Of the installed medical imaging equipment in the country (excluding ultrasound systems) this study found general X-ray as the most dominant, representing 67% of the surveyed systems and 14.4 u/mp. Each of the regions in Ghana has installed X-ray systems and these are routinely used as a common diagnostic imaging tool to support healthcare delivery in clinical cases such as bone fractures, arthritis in joints and chest conditions like tuberculosis. General X-ray systems are common in the country due to their ease of use, low cost, minimal maintenance as well as the relatively low-level radiation exposure associated with the procedure [21]. As part of a national programme for detection of tuberculosis (TB) cases in Ghana, the government in 2016 installed 52 X-ray systems across the country [22]. In addition to TB diagnosis, these systems have found use for a variety of other health needs and largely contributed to strengthening the Ghanaian healthcare system.

The private sector, constituting 52% of the medical imaging centres in Ghana (Table 1), has made immense contribution to diagnostic imaging healthcare delivery. This is perceived to be laudable in healthcare provision in a country that sees the private sector as the engine of growth. The Greater Accra, Eastern and Bono regions, with imaging units per medical centre ratios of 3.0, 3.0 and 3.3 respectively, were found greater than the national average of 2.5. The ratios of Eastern and Bono regions are found to be high due to low numbers of diagnostic medical facilities in these regions in relation to the corresponding installed imaging equipment.

As presented in Table 3, the national estimates for equipment/population ratios ranged from 14.4 u/mp for general X-rays to 0.03 u/mp for SPECT gamma camera. None of the available imaging systems measure above WHO´s recommended equipment units per million population. Coupled with this observation is the skewness in regional distribution of the installed imaging equipment towards the Greater Accra and Ashanti regions which accounts for 58% of all imaging systems nationally. The two regions, being the most populous and highly cosmopolitan in nature, naturally attract a lot more of national infrastructure and businesses from both public and the private sector. A great deal of effort is anticipated from government to put in required measures to bridge the gap by increasing availability of radiological health services in other parts of the country and promote equitability in medical imaging infrastructure at the regional level.

The regional estimates of equipment/population ratios in decreasing order per this study is Greater Accra (49.6 u/mp), Ashanti (22.4 u/mp), Western (21.4 u/mp), Eastern (20.6 u/mp), Bono East (20.0 u/mp), Bono (19.2 u/mp), Volta (17.9 u/mp), Upper West (16.7 u/mp), Oti (12.5 u/mp), Central (11.9 u/mp), Northern (8.9 u/mp), Ahafo (8.9 u/mp), Upper East (6.9 u/mp), Western North (6.7 u/mp), Savannah (5.5 u/mp) and North-East (1.7 u/mp). The regions with equipment/population density above 20 u/mp are in line with the urbanized and cosmopolitan nature of such places. On the national scale, the imaging modalities did not meet their corresponding OECD average equipment/population ratios for CT (25.72), mammography (24.34) and MRI (15.94).

Ghana´s equipment/population ratio relative to South Africa indicates a better medical imaging infrastructure per unit population in the latter, primarily as a result of its higher economic strength. South Africa´s gross domestic product (GDP) per capita of 5,067 USD is three-fold that of Ghana (1,700 USD) and this is known to have a direct correlation on health infrastructure. Relatively, Ghana is also seen to perform better in equipment/population ratio compared to countries like Kenya, Tanzania, Uganda, Zambia and Zimbabwe, however, the country falls below the recommended WHO target and OECD averages [19,20]. A major challenge in each of the African countries will be to address the geographical discrepancies in distribution of imaging equipment [13-17]. If Ghana is to achieve equitable access to diagnostic imaging, the least-resourced regions of the country will have to be afforded priority when allocating future equipment resources. A constraint to achieving OECD-level imaging capacity in Ghana and other African countries is the huge discrepancy in health expenditure per capita between countries in the OECD region and those in Africa. OECD countries have always had a culture of investing more resources to cater for radiology services for their population.

Published data on radiological health facilities in Ghana has been scanty. With the few available data, much of the information is largely out-of-date. Schandorf and Tetteh [23] and RAD-AID [21] highlight the structure of radiology services in Ghana, as pertaining in the late 1990s, as follows: 1) University teaching hospitals, with specialist radiologists and radiographers, and operate radiology departments in conjunction with the medical schools. All medical imaging equipment including general X-rays, CT, dental X-rays, fluoroscopy, angiography, MRI, etc. are available; 2) regional hospitals, with radiological services operated by radiographers under the supervision of senior medical officers in charge of the hospital. Basic equipment is mostly available for radiological examinations; 3) district hospitals, with about 60% having basic radiological equipment available for conventional radiography operated by trained senior technical officers under the supervision of senior medical officers in charge; 4) district health posts and centres, mostly with no imaging services; 5) service agency hospitals, established by agencies like the police, military and social security agencies. Such facilities offer conventional radiography and fluoroscopy.

In recent times, the categories of health facilities have seen sophistication in diagnostic equipment, with district, regional and some private hospitals and imaging centres installing systems such as CT, MRI and fluoroscopy. Ghana health service, the technical wing of the ministry of health, has introduced telemedicine schemes, but currently do not include diagnostic radiology reporting due to limited availability of Picture Archiving Communication Systems (PACS) in the health facilities. Only a few of the radiology centres have installed PACS systems, permitting intra- and inter-facility telemedicine procedures. The country also lacks local manufacturing of radiology equipment and parts, a cause for delay in importation of parts for broken equipment and high maintenance costs.

The strength of this study has been the identification of gap in diagnostic imaging infrastructure in Ghana, which directly translates into geographical access to radiological services. This study would inform government in terms of future planning of budget for healthcare delivery in the country. The study is however limited in its exclusion of diagnostic ultrasound data.

## Conclusion

This paper has outlined radiological imaging equipment availability in Ghana in all its 16 regions. Disparities in the distribution of medical imaging equipment in the country is glaringly projected by this study and a call for steps by government to homogenize the distribution and to bridge the gap, as per WHO recommendation, thereby creating equitable access to radiological services nationally. The private sector´s contribution to diagnostic imaging services is laudable and should be further encouraged.

### What is known about this topic

- Audit of registered diagnostic radiology equipment resources have already been conducted in at least five African countries (South Africa, Tanzania, Uganda, Zambia, Zimbabwe);
- The WHO recommends equitable distribution of diagnostic imaging equipment nationally and at the global level for improved healthcare delivery.

### What this study adds

- The study is the foremost comprehensive audit of diagnostic imaging equipment resources conducted in Ghana;
- There exists a wide disparity in the distribution of medical imaging equipment between different geographical regions of Ghana;
- Ghana´s diagnostic unit/population ratio is below the OECD average and WHO´s recommendation.
